# Evaluation of the relationship between idiopathic restless legs syndrome and serum hepcidin levels

**DOI:** 10.1002/brb3.3259

**Published:** 2023-09-19

**Authors:** Semra Alaçam Köksal, Sena Boncuk Ulaş, Bilgehan Atılgan Acar, Türkan Acar, Yeşim Güzey Aras, Mehmet Köroğlu

**Affiliations:** ^1^ Department of Neurology Kocaeli İzmit SEKA State Hospital İzmit Kocaeli Turkey; ^2^ Department of Neurology Keşan State Hospital Edirne Turkey; ^3^ Department of Neurology, Faculty of Medicine Sakarya University Serdivan Sakarya Turkey; ^4^ Department of Microbiology, Faculty of Medicine Sakarya University Serdivan Sakarya Turkey

**Keywords:** iron, iron deficiency, movement disorders, restless legs syndrome, serum hepcidin

## Abstract

**Introduction:**

The relationship between restless legs syndrome (RLS) and iron deficiency is a well‐known topic. However, the etiology of the disease has not been determined. As the central iron deficiency is the most critical biological abnormality for RLS, we planned a study examining the relationship between RLS and hepcidin, which is the only regulatory hormone of iron metabolism known so far.

**Methods:**

International Restless Legs Syndrome Study Group diagnostic criteria (2014) were taken as a basis. A total of 40 RLS patients and 40 healthy controls were included in the study. To avoid the potential variables that might cause secondary RLS, all the participants were asked to provide hemogram, ferritin, iron, thyroid function tests, and sedimentation analysis. The hepcidin levels were measured with a Human Hepcidin (Hepc 25) ELISA kit (MyBioSource).

**Results:**

The statistically significant results of our analysis show that the red blood cell count, the neutrophil count, the percentage of lymphocytes and neutrophils, and, more distinctively, hepcidin levels were higher in RLS patients in comparison with the control group.

**Conclusion:**

In this study, no differences were found in iron and ferritin values. High levels of hepcidin, the main regulator of iron metabolism, in those with primary RLS support the possibility that hepcidin may play a role in the pathogenesis of RLS. We think that larger studies on this subject can give clearer ideas.

## Graphical abstract

• Iron mechanisms are held responsible for the pathogenesis of RLS.

• The serum hepcidin level is higher in Restless Legs Syndrome patients.

• Hepcidin may be a marker that is involved in iron metabolism and also plays a role in RLS.

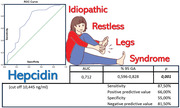

## 1 INTRODUCTION

Restless legs syndrome (RLS) is a movement disorder that affects the sensorimotor system and is characterized by the feeling of discomfort and desire to move, especially in the lower extremities, which often occurs at night, and the relief of symptoms with movement (Allen, Picchietti et al., 2014; Klingelhoefer et al., 2016; Manconi et al., 2021). RLS can be seen as idiopathic (primary) or secondary. Although the secondary form has been associated frequently with iron deficiency (Chokroverty, 2015), the etiology of idiopathic RLS has not been fully elucidated. Studies on the pathophysiology of RLS have focused on genetics, brain iron metabolism, dysfunction in dopaminergic pathways, and glutamate and adenosine‐related changes (Ondo, 2014; Weinstock et al., 2012).

It is well known that iron deficiency predisposes to RLS in both idiopathic and secondary forms. Nordlander (1954) first described the relationship between RLS and serum iron (SI) deficiency. He hypothesized that due to the presence of RLS patients with sufficient iron levels in the peripheral blood, iron deficiency in tissues is possible despite normal SI (Nordlander, 1954). This has prompted researchers to investigate whether there is central iron deficiency in RLS patients when SI is sufficient. In the following years, central iron deficiency in idiopathic RLS was documented by cerebrospinal fluid, magnetic resonance imaging, and autopsy studies (Allen et al., 2001; Clardy et al., 2006; Mizuno et al., 2005).

Although the discovery of hepcidin, a peptide hormone that is predominantly expressed in the liver in human studies and released from the choroid plexus in animal models, initially attracted attention due to its antimicrobial properties, its effects on iron metabolism were quickly understood, and this was a breakthrough in the pathophysiology and diagnosis of iron metabolism–related diseases (Krause et al., 2000; Park et al., 2001; Urrutia et al., 2013). Hepcidin level increases in the case of increased body iron level and infection‐inflammation and decreases in hypoxia, anemia, and erythropoiesis. Increased hepcidin binds to the ferroportin‐1, iron exporter, and inhibits iron release from enterocytes, macrophages, and hepatocytes (Silva & Faustino, 2015).

Because central iron deficiency is the most critical biological abnormality proven for RLS and chronic inflammation is blamed for the etiology of RLS (Weinstock et al., 2012), we planned a study examining the relationship between RLS and hepcidin, which is the only regulatory hormone of iron known so far, and the synthesis of which is also regulated by inflammation. Although there are studies on RLS and hepcidin in the literature in recent years, the number of studies on the relationship between idiopathic RLS and hepcidin is very few. As a result of our study, we aimed at contributing to the literature on the role of hepcidin in RLS pathogenesis.

## 2 MATERIALS AND METHODS

Patients who applied to the neurology clinic of Sakarya University Training and Research Hospital and were diagnosed with idiopathic RLS according to the consensus criteria of the International RLS Study Group (IRLSSG) published in 2014 (Allen, Picchietti et al., 2014);, did not have any other disease, did not use medication, had normal neurological examinations, and were over 18 years of age were included in the study. As the control group, 40 volunteers who applied to the hospital for blood donation were matched with the patient group in terms of age–sex and did not have a known disease or drug use included. The World Medical Association (WMA) Helsinki Declaration was complied with in all stages of this study. Approval was obtained from the Sakarya University Clinical Research Ethics Committee before starting the study. The written informed consent form was obtained from all participants. In both groups, conditions that could lead to secondary RLS, such as pregnancy, diabetes mellitus, end‐stage renal failure, iron deficiency and iron deficiency anemia, rheumatological diseases, multiple sclerosis, neurological diseases such as Parkinson's disease, hypo‐hyperthyroidism, and the use of drugs that could cause RLS, were excluded.

RLS severity was measured with the disease severity scale determined by the IRLSSG. Body mass indexes (BMIs) of the patient and control groups, disease duration, and family history of the patient group were noted. In this study, serum hepcidin level, complete blood count, ferritin, SI, comprehensive biochemistry, thyroid function tests, and erythrocyte sedimentation rate were studied for all participants.

Venous blood samples of all patients were taken in the morning after the overnight fast. Complete blood counts were studied immediately by placing 2 cm^3^ of blood into a tube containing EDTA. Complete blood count parameters were measured with a CELL‐DYN 3700 blood counter (Abbott Diagnostics). According to WHO criteria, hemoglobin values below 12 mg/dL in women and 13 mg/dL in men were considered anemia (Beutler & Waalen, 2006). For serum samples, 6 cm^3^ of blood was taken into an empty tube, prepared at room temperature for 30 min, then centrifuged at 4000 rpm for 10 min. All serum samples were stored at −80°C until working in the laboratory. SI and other biochemistry parameters were analyzed by photometric method with an Abbott Architect C16000 (Abbott Diagnostics) device. Our laboratory's normal range of SI levels was 40–145 μg/dL for women and 65–175 μg/dL for men. Serum ferritin and TSH values were analyzed by chemiluminescent microparticle immunoassay using the Abbott Architect I 2000 (Abbott Diagnostics) device. The normal range for serum ferritin in our laboratory was 4.63–204 ng/mL in women and 21.81–274 ng/mL in men. Patients whose SI and ferritin values were not within the specified range and patients with anemia were excluded from the study. Hepcidin was measured with the Human Hepcidin 25 (Hepc25) ELISA kit (MyBioSource; Cat No: MBS269929) by the manufacturer's instructions. The detection range for the kit was 200–3.12 ng/mL. The lowest detectable human hepcidin 25 level was 0.6 ng/mL.

Statistical evaluation was performed using the SPSS (Statistical Package for Social Sciences) 21.0 package program. Mean, standard deviation, median, lowest and highest value, frequency, and ratio values were used in the descriptive statistics of the data. The Kolmogorov–Smirnov test measured whether the distribution of continuous and discrete numerical variables conformed to normal distribution. Mann–Whitney *U* test and independent sample *t*‐test were used to analyze quantitative data. The chi‐square test was used in the analysis of categorical data. The effect level and cut‐off value were investigated with the receiver operating characteristic (ROC) curve. The relationship among hepcidin and hemoglobin, ferritin, and iron was evaluated by the Spearman correlation analysis. The effect level was analyzed by univariate and multivariate logistic regression. The statistical significance level was accepted as *p* < .05.

## 3 RESULTS

The study included 30 females and 10 males with a mean age of 46.85 ± 8.6 years, 40 RLS patients, and 26 females and 14 males with a mean age of 44.83 ± 9.8 years, 40 healthy controls. The age, gender distribution, and BMI values of the patients in the patient and control groups did not show a statistically significant difference. Although the mean disease duration in the RLS group was 11.1 ± 6.8 years, the mean time after diagnosis was 3.2 years. In the patient group, 36 people had a positive family history for RLS. In terms of RLS severity, the mean score in the patient group was 25.4 ± 6.8 (Table 1).

**TABLE 1:** Demographic and clinical characteristics of restless legs syndrome (RLS) patients and control group.

		Patient group			Control group			*P*
		Min–max	Median	Mean ± sd/*n* (%)	Min–max	Median	Mean ± sd/*n* (%)	
Age		27–64	48	46.9 ± 8.6	27–61	46	44.8 ± 9.8	.330*^t^*
Gender	Female		30	75%		26	65%	.329^χ^ ^2^
	Male		10	25%		14	35%	
BMI		20–48	27	28.5 ± 5.7	16–45	28	28.4 ± 5.6	.795*^m^*
	Underweight		0	0%		1	3%	
	Normal		10	25%		6	15%	
	Overweight		20	50%		19	48%	
	Obese		8	20%		12	30%	
	Morbid obesity		2	5%		2	5%	
Duration of illness (years)			11.1 ± 6.8					
Duration of diagnosis (years)			3.2					
RLS family history (yes/no)			26/14			0/40		
IRLSSG severity score		10–36	25	25.4 ± 6.8				

*Note*: ^t^, independent sample *t* test; *^χ^* ^2^, chi‐square test; *^m^*, Mann–Whitney *U* test.

Abbreviations: IRLSSG, International Restless Legs Syndrome Study Group; SD, standard deviation.

Ferritin, iron, white blood cell, hemoglobin, hematocrit, platelet values, lymphocyte count, percentage of monocytes, and monocyte count did not differ significantly between the patient and control groups. Red blood cell count, neutrophil count, lymphocyte percentage, and, more particularly, neutrophil percentage and hepcidin values were significantly higher in the patient group than in the control group (*p* ˂ .05) (Table 2).

**TABLE 2:** Laboratory results of the patient and control group.

	Patient group			Control group			*pm*
	Min–max	Median	Mean ± sd/*n* (%)	Min–max	Median	Mean ± sd/*n* (%)	
Ferritin (ng/mL)	7–167	39	46.7 ± 38.7	7–154	36	49.9 ± 39.0	.607
Iron (μg/dL)	40–195	79	85.9 ± 36.1	50–208	86	96.6 ± 36.5	.09
WBC	3.5–11.7	6.9	7.0 ± 1.8	1.4–11.7	5.8	6.5 ± 2.0	.186
RBC	4.1–5.8	4.7	4.7 ± 0.3	3.8–5.8	4.5	4.6 ± 0.5	***.031***
Hemoglobin (g/dL)	12–16	14	13.7 ± 1.1	12–17	14	13.7 ± 1.4	.84
Hematocrit	34–48	40	40.2 ± 3.2	34–51	39	40.0 ± 4.1	.535
Platelet	178–464	271	276 ± 73	191–447	279	282 ± 57	.45
Neu (%)	47–71	59	58.6 ± 6.6	39–74	55	54.0 ± 7.4	***.008***
Neu	1.7–7.8	4	4.1 ± 1.3	0.7–6.5	3.1	3.5 ± 1.3	***.021***
Lym (%)	20–43	31	31.1 ± 6.1	20–49	34	35.0 ± 6.7	***.013***
Lym	1.1–3.8	2.0	2.1 ± 0.6	0.5–4.5	2.2	2.3 ± 0.8	.567
Monocytes (%)	2.8–10.3	7.0	6.7 ± 1.7	3.1–10.9	7	6.6 ± 1.8	.564
Monocytes	0.2–1.0	0.5	0.5 ± 0.1	0.1–0.7	0.4	0.4 ± 0.1	.292
Hepcidin (ng/mL)	10–14	11	11.5 ± 0.9	10–15	11	11.0 ± 1.0	***.001***

*Note*: ^m^, Mann–Whitney *U* test.

Abbreviations: WBC: White Blood Cell RBC: Red Blood Cell. Statistically significant p<0.05 values are shown in bold and italic.

**FIGURE 1:**
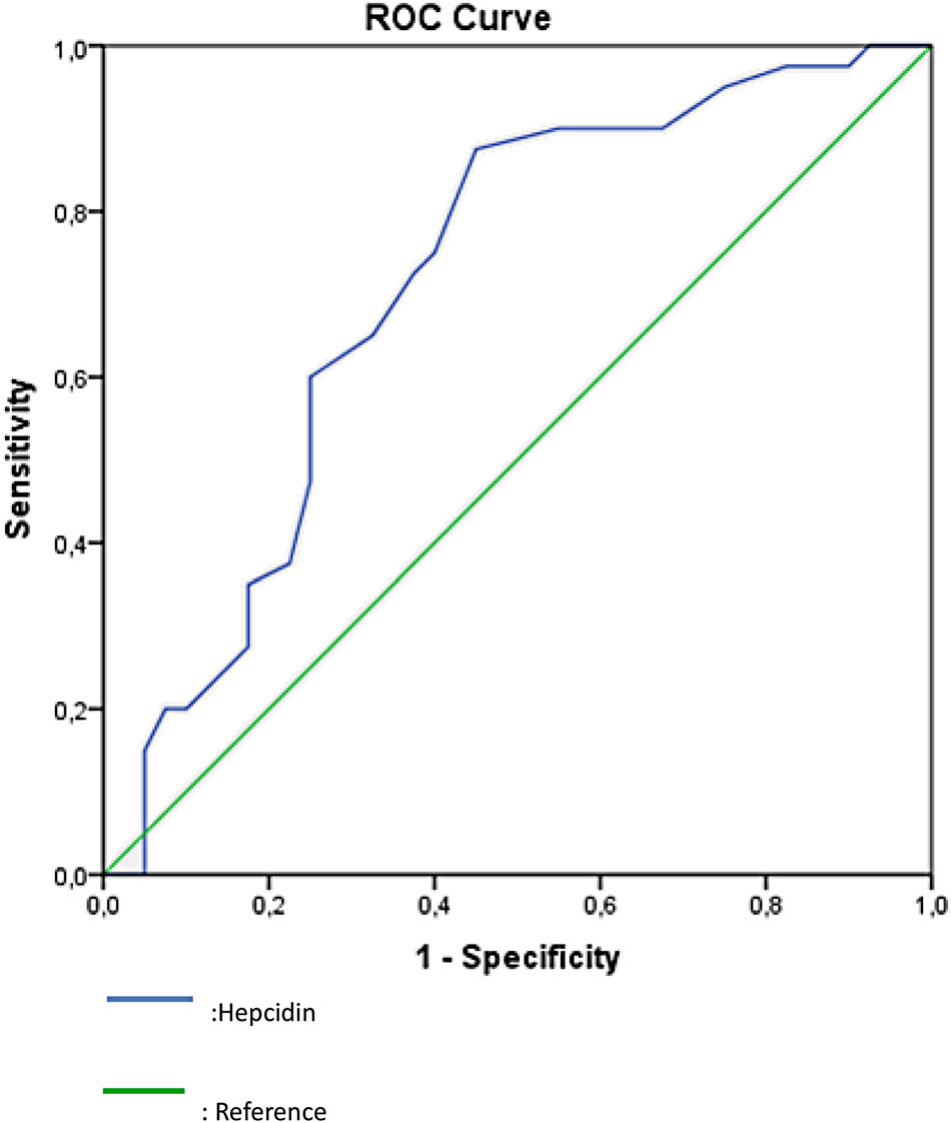
ROC (Receiver operating characteristic) analysis of the relation between serum hepcidin levels and restless legs syndrome (RLS). A significant predictive effect of hepcidin level was observed in the differentiation of patient and control groups.

In the univariate model, neutrophil and lymphocyte percentage and hepcidin level were observed in the separation of the patient and control groups (*p* ˂ .05). In addition to that, in the multivariate regression model, hepcidin level was observed in the distinction between the patient and control groups (*p* ˂ .05). A significant predictive effect of hepcidin level was found in the differentiation of patient and control groups. The highest hepcidin value (cut‐off value) was 10.45 ng/mL in the area under the curve (AUC: 0.712 [0.596–0.828]) in the differentiation of patient and control groups. Sensitivity was 87.5%; the positive predictive value was 66%; specificity was 55%; the negative predictive value was 81.5% (Figure 1, Table 3).

**TABLE 3:** ROC (Receiver operating characteristic) analysis of the relation of serum hepcidin levels and restless legs syndrome (RLS).

	AUC	%95 CI	*P*
Hepcidin (cut off 10,445 ng/mL)	0.712	0.596–0.828	***.001***
		Sensitivity	87.50%
		Positive predictive value	66.00%
		Specificity	55.00%
		Negative predictive value	81.50%

Abbreviation: AUC, area under the curve. CI: Confidence Interval. Statistically significant p<0.05 values are shown in bold and italic.

In the correlation analysis between hepcidin and iron, the *r* value was found to be −.263, and there was a statistically significant weak negative correlation found (*p*: .018). There was a weak negative correlation between hepcidin and hemoglobin and ferritin, and this correlation was not statistically significant (*p* > .05).

## 4 DISCUSSION

The prominent result of our study is that serum hepcidin level, which plays an important role in iron metabolism, is significantly higher in idiopathic RLS patients compared to healthy controls. It has been known since the 1900s that iron metabolism disorder plays a role in the pathophysiology of RLS. Iron deficiency is currently the best proven risk factor for secondary RLS (Nordlander, 1954). Moreover, aggressive iron deficiency treatment has been reported to reduce the severity of RLS in these cases. However, most RLS patients have normal serum ferritin, and their peripheral iron stores seem sufficient. This suggests that RLS is related to central nervous system iron status instead of peripheral (Allen, 2015; Trenkwalder et al., 2016).

In recent years, the number of studies investigating markers that may be associated with RLS has been increasing. Although RLS has been a known disease for many long years, the diagnosis is predominantly made clinically, and the absence of a method to confirm the diagnosis causes underdiagnoses or misdiagnoses (Bellei et al., 2018). It is seen that some of the studies focused on RLS‐related inflammatory factors such as CRP and neutrophil–lymphocyte ratio, whereas another part focused on iron metabolism factors (Jiménez‐Jiménez et al., 2023; Karadeniz et al., 2023). The underlying mechanism of reduced iron concentration in the brains of RLS patients is not yet understood. Still, studies support that the source of brain iron deficiency is the blood–brain barrier interface (Dauvilliers & Winkelmann, 2013). Blood–brain barrier epithelial cells serve as the brain's iron reservoir. In RLS, a loss of iron‐regulating protein activity in the microcirculation may cause the impairment of iron transport to the blood–brain barrier and the insufficiency of iron stores in endothelial cells (Connor et al., 2011). In a study conducted with 12 early, 13 late‐onset idiopathic RLS patients, and a control group of 14 in which pro‐hepcidin levels were measured in the CSF (Cerebrospinal Fluid), pro‐hepcidin levels in the CSF of early‐onset RLS patients were reported to be significantly decreased compared to the control group. In the autopsy examination performed with eight idiopathic RLS patients and a control group consisting of eight people, it was reported that the concentration of pro‐hepcidin in the neuromelanin cells, substantia nigra, and putamen of the patients with RLS increased compared to the control group. In the same study, immunohistochemical evidence was obtained indicating that ferroportin was secreted in ependymal cells surrounding the ventricles and in choroid plexus epithelial cells. In this case, it could not be differentiated whether the decrease in pro‐hepcidin levels in the CSF occurs in response to the decrease in brain iron or is the result of faulty signaling in RLS (Clardy et al., 2006).

Data suggest that immune‐inflammatory mechanisms may play a role in some patients evaluated for idiopathic RLS. Chronic inflammation that may be responsible for RLS pathophysiology may lead to increased circulating hepcidin levels. IL‐6, which occurs in inflammation, can stimulate hepcidin production, and hypoxia and LPS (lipopolysaccharide) production also increase hepcidin levels. Increased hepcidin may also bind to the choroid plexus‐related ferroportin, reducing the amount of iron available for the central nervous system (Weinstock et al., 2012). We included patients with normal WBC (white blood cell) and erythrocyte sedimentation rate values in our patients and control group to rule out the active inflammatory process. Although secondary causes such as anemia and inflammatory processes were excluded, the red blood cell value, neutrophil count and percentage, lymphocyte percentage, and hepcidin value were significantly higher in RLS patients compared to the control group.

In our study, the mean age of RLS patients was 46.85 ± 8.6 (years). The late diagnosis of RLS may be due to the fact that the patients downplayed their complaints when their symptoms were mild at the beginning, consulted the doctor late and the doctors did not have enough information about RLS. The patients with RLS had worse sleep quality and were more depressed, in line with the literature (Allen, Chen et al., 2014; Picchietti & Winkelman, 2005).

In our study, SI levels and ferritin levels, indicators of iron metabolism in the peripheral circulation, were evaluated, and patient controls within normal limits were included. In this way, secondary etiologies related to peripheral iron deficiency were ruled out. In the study conducted by Dauvilliers et al. (2018), serum hepcidin level in idiopathic RLS patients without drug use was higher than in the control group, as in our study. In a study conducted with RLS patients with chronic hemodialysis disease, serum hepcidin levels were found to be higher than in the control group; in addition, there was a positive correlation between RLS severity and serum hepcidin levels (Tufekci & Kara, 2021). In another study conducted in 2020, there was no significant difference in the levels of hepcidin among patients who had not been treated for RLS and controls, but there were 18 patients and 15 healthy control groups and the number of patients was very low (Im et al., 2020). In the study of 466 RLS and 9242 control groups from Danish Blood Donors, there was no significant difference in serum hepcidin levels between groups, but RLS patients were not classified as idiopathic or secondary (Dowsett et al., 2021).

In the study conducted with 2998 participants, who investigated the relationship between serum hepcidin value reference ranges and serum hepcidin values in biochemical parameters in the general population, serum hepcidin concentration in men does not change with age and is in the reference range of 1674–65,007 ng/mL; It is in the reference range of 1116–54,963 ng/mL in women under the age of 55, and 3348–69,112 ng/mL in women aged 55 and over. Although the mean value in men did not change with age, it was higher in postmenopausal women than in women in the premenopausal period. In the same study, serum hepcidin values in the morning (before 12:00 p.m.) were lower than in the evening (after 5:00 p.m.) for both genders. A positive correlation was found between serum ferritin values and serum hepcidin values (Galesloot et al., 2011). Our study showed no statistically significant correlation between serum ferritin and serum hepcidin values. In contrast, there was a weak negative correlation between SI and serum hepcidin levels. The reason for this difference is that in our study, unlike the general population, secondary RLS causes were excluded, that is, we worked with a population without peripheral iron deficiency. In our study, the cut‐off value of hepcidin for ROC diagnosis was found to be 10.45 ng/ml in the analysis performed using the ROC curve to investigate the sensitivity and specificity in diagnosing RLS (Table 3).

In the study by Chenini et al., hepcidin level was higher in RLS patients with normal ferritin levels compared to the control group. In the same study, high hepcidin levels were associated with older age, older age at RLS onset, less daytime sleepiness, and idiopathic RLS. They reported that hepcidin levels in RLS patients were higher independent of treatment and history of augmentation (Chenini et al., 2020).

In conclusion, no differences were found in iron and ferritin values. High levels of hepcidin, the main regulator of iron metabolism, those with primary RLS support the possibility that hepcidin may play a role in the pathogenesis of RLS. In our study, the lack of cerebrospinal fluid examination to compare the central nervous system and peripheral iron status, and the inability to obtain morning and evening serum hepcidin levels separately due to the circadian rhythm feature are important shortcomings. We think that larger studies on this subject, in which cerebrospinal fluid measurements are also carried out, can give clearer ideas about the relation between hepcidin and RLS.

## AUTHOR CONTRIBUTIONS

**Semra Alaçam Köksal**: Formal analysis; methodology; project administration; resources; writing—original draft; writing—review and editing. **Sena Boncuk Ulaş**: Data curation; formal analysis; methodology; software; writing—original draft; writing—review and editing. **Bilgehan Atılgan Acar**: Formal analysis; supervision; writing—original draft; writing—review and editing. **Türkan Acar**: Data curation; investigation; resources; visualization; writing—review and editing. **Yeşim Güzey Aras**: Conceptualization; data curation; resources; visualization. **Mehmet Köroğlu**: Data curation; investigation; resources; visualization.

## CONFLICT OF INTEREST STATEMENT

There are no personal or financial conflicts of interest in the article.

### PEER REVIEW

The peer review history for this article is available at https://publons.com/publon/10.1002/brb3.3259.

## Data Availability

The data are not publicly available due to privacy or ethical restrictions; research data are not shared.

## References

[brb33259-bib-0001] Allen, R. P. (2015). Restless leg syndrome/Willis‐Ekbom disease pathophysiology. Sleep Medicine Clinics, 10(3), 207–214. xi. 10.1016/j.jsmc.2015.05.022. Epub 2015 Jul 15. 26329430 PMC4559751

[brb33259-bib-0002] Allen, R. P. , Barker, P. B. , Wehrl, F. , Song, H. K. , & Earley, C. J. (2001). MRI measurement of brain iron in patients with restless legs syndrome. Neurology, 56(2), 263–265. 10.1212/wnl.56.2.263. Erratum in: Neurology. 2015 Jan 6;84(1):105. Wehrl, F [corrected to Wehrl, F W]. 11160969

[brb33259-bib-0003] Allen, R. P. , Chen, C. , Garcia‐Borreguero, D. , Polo, O. , Dubrava, S. , Miceli, J. , Knapp, L. , & Winkelman, J. W. (2014). Comparison of pregabalin with pramipexole for restless legs syndrome. New England Journal of Medicine, 370(7), 621–631. 10.1056/NEJMoa1303646 24521108

[brb33259-bib-0004] Allen, R. P. , Picchietti, D. L. , Garcia‐Borreguero, D. , Ondo, W. G. , Walters, A. S. , Winkelman, J. W. , Zucconi, M. , Ferri, R. , Trenkwalder, C. , & Lee, H. B. , International Restless Legs Syndrome Study Group . (2014). Restless legs syndrome/Willis‐Ekbom disease diagnostic criteria: Updated International Restless Legs Syndrome Study Group (IRLSSG) consensus criteria–history, rationale, description, and significance. Sleep Medicine, 15(8), 860–873. 10.1016/j.sleep.2014.03.025. Epub 2014 May 17. 25023924

[brb33259-bib-0005] Bellei, E. , Monari, E. , Ozben, S. , Koseoglu Bitnel, M. , Topaloglu Tuac, S. , Tomasi, A. , & Bergamini, S. (2018). Discovery of restless legs syndrome plasmatic biomarkers by proteomic analysis. Brain and Behavior, 8, e01062. 10.1002/brb3.1062 30244532 PMC6192389

[brb33259-bib-0006] Beutler, E. , & Waalen, J. (2006). The definition of anemia: What is the lower limit of normal of the blood hemoglobin concentration? Blood, 107(5), 1747–1750. 10.1182/blood-2005-07-3046. Epub 2005 Sep 27. 16189263 PMC1895695

[brb33259-bib-0007] Chenini, S. , Delaby, C. , Rassu, A.‐L. , Barateau, L. , Vialaret, J. , Hirtz, C. , Dupuy, A. M. , Lehmann, S. , Jaussent, I. , & Dauvilliers, Y. (2020). Hepcidin and ferritin levels in restless legs syndrome: A case–control study. Scientific Reports, 10(1), 11914. 10.1038/s41598-020-68851-0 32681031 PMC7367854

[brb33259-bib-0008] Chokroverty, S. (2015). Differential diagnoses of restless legs syndrome/Willis‐Ekbom disease: Mimics and comorbidities. Sleep Medicine Clinics, 10(3), 249–262. xii. 10.1016/j.jsmc.2015.05.021 26329435

[brb33259-bib-0009] Clardy, S. L. , Wang, X. , Boyer, P. J. , Earley, C. J. , Allen, R. P. , & Connor, J. R. (2006). Is ferroportin‐hepcidin signaling altered in restless legs syndrome? Journal of the Neurological Sciences, 247(2), 173–179. 10.1016/j.jns.2006.04.008. Epub 2006 Jun 8. 16759669

[brb33259-bib-0010] Connor, J. R. , Ponnuru, P. , Wang, X.‐S. , Patton, S. M. , Allen, R. P. , & Earley, C. J. (2011). Profile of altered brain iron acquisition in restless legs syndrome. Brain, 134(Pt 4), 959–968. 10.1093/brain/awr012. Epub 2011 Mar 11. 21398376 PMC3069701

[brb33259-bib-0011] Dauvilliers, Y. , Chenini, S. , Vialaret, J. , Delaby, C. , Guiraud, L. , Gabelle, A. , Lopez, R. , Hirtz, C. , Jaussent, I. , & Lehmann, S. (2018). Association between serum hepcidin level and restless legs syndrome. Movement Disorders, 33(4), 618–627. 10.1002/mds.27287. Epub 2018 Feb 8. 29418021

[brb33259-bib-0012] Dauvilliers, Y. , & Winkelmann, J. (2013). Restless legs syndrome: Update on pathogenesis. Current Opinion in Pulmonary Medicine, 19(6), 594–600. 10.1097/MCP.0b013e328365ab07 24048084

[brb33259-bib-0013] Dowsett, J. , Didriksen, M. , Larsen, M. H. , Burgdorf, K. S. , Thørner, L. W. , Sørensen, E. , Erikstrup, C. , Pedersen, O. B. , Ostrowski, S. R. , & Ullum, H. (2021). No association between plasma hepcidin levels and restless legs syndrome—Results from the Danish Blood Donor study. Sleep Medicine, 88, 68–73. 10.1016/j.sleep.2021.10.008. Epub 2021 Oct 15. 34736065

[brb33259-bib-0014] Galesloot, T. E. , Vermeulen, S. H. , Geurts‐Moespot, A. J. , Klaver, S. M. , Kroot, J. J. , Van Tienoven, D. , Wetzels, J. F. M. , Kiemeney, L. A. L. M. , Sweep, F. C. , Den Heijer, M. , & Swinkels, D. W. (2011). Serum hepcidin: Reference ranges and biochemical correlates in the general population. Blood, 117(25), e218–e225. 10.1182/blood-2011-02-337907 21527524

[brb33259-bib-0015] Im, H.‐J. , Kim, J. H. , Yun, C.‐H. , Kim, D. W. , & Oh, J. (2020). Changes in hepcidin serum levels correlate with clinical improvement in idiopathic restless legs syndrome patients. Journal of Clinical Medicine, 9(12), 4115. 10.3390/jcm9124115 33419264 PMC7766726

[brb33259-bib-0016] Jiménez‐Jiménez, F. J. , Alonso‐Navarro, H. , García‐Martín, E. , & Agúndez, J. A. G. (2023). Inflammatory factors and restless legs syndrome: A systematic review and meta‐analysis. Sleep Medicine Reviews, 68, 101744. 10.1016/j.smrv.2022.101744. Epub 2022 Dec 31. 36634410

[brb33259-bib-0017] Karadeniz, M. , Ser, M. H. , Nalbantoglu, M. , Tumay, F. B. , Yilmaz, N. , Acikgoz, S. , & Senel, G. B. (2023). Neutrophil‑to‑lymphocyte ratio as a marker of inflammation in restless legs syndrome during pregnancy. Bratislavske Lekarske Listy, 124(1), 42–46. 10.4149/BLL_2023_006 36519606

[brb33259-bib-0018] Klingelhoefer, L. , Bhattacharya, K. , & Reichmann, H. (2016). Restless legs syndrome. Clinical Medicine (London), 16(4), 379–382. 10.7861/clinmedicine.16-4-379 PMC6280211 27481386

[brb33259-bib-0019] Krause, A. , Neitz, S. , Mägert, H.‐J. , Schulz, A. , Forssmann, W.‐G. , Schulz‐Knappe, P. , & Adermann, K. (2000). LEAP‐1, a novel highly disulfide‐bonded human peptide, exhibits antimicrobial activity. FEBS Letters, 480(2–3), 147–150. 10.1016/s0014-5793(00)01920-7 11034317

[brb33259-bib-0020] Manconi, M. , Garcia‐Borreguero, D. , Schormair, B. , Videnovic, A. , Berger, K. , Ferri, R. , & Dauvilliers, Y. (2021). Restless legs syndrome. Nature Reviews Disease Primers, 7(1), 80. 10.1038/s41572-021-00311-z 34732752

[brb33259-bib-0021] Mizuno, S. , Mihara, T. , Miyaoka, T. , Inagaki, T. , & Horiguchi, J. (2005). CSF iron, ferritin and transferrin levels in restless legs syndrome. Journal of Sleep Research, 14(1), 43–47. 10.1111/j.1365-2869.2004.00403.x 15743333

[brb33259-bib-0022] Nordlander, N. B. (1954). Restless legs. British Journal of Physical Medicine, 17(7), 160–162. 13172443

[brb33259-bib-0023] Ondo, W. G. (2014). Restless legs syndrome: Pathophysiology and treatment. Current Treatment Options in Neurology, 16(11), 317. 10.1007/s11940-014-0317-2 25238731

[brb33259-bib-0024] Park, C. H. , Valore, E. V. , Waring, A. J. , & Ganz, T. (2001). Hepcidin, a urinary antimicrobial peptide synthesized in the liver. Journal of Biological Chemistry, 276(11), 7806–7810. 10.1074/jbc.M008922200. Epub 2000 Dec 11. 11113131

[brb33259-bib-0025] Picchietti, D. , & Winkelman, J. W. (2005). Restless legs syndrome, periodic limb movements in sleep, and depression. Sleep, 28(7), 891–898. 16124671

[brb33259-bib-0026] Silva, B. , & Faustino, P. (2015). An overview of molecular basis of iron metabolism regulation and the associated pathologies. Biochimica et Biophysica Acta, 1852(7), 1347–1359. 10.1016/j.bbadis.2015.03.011. Epub 2015 Apr 2. 25843914

[brb33259-bib-0027] Trenkwalder, C. , Allen, R. , Högl, B. , Paulus, W. , & Winkelmann, J. (2016). Restless legs syndrome associated with major diseases: A systematic review and new concept. Neurology, 86(14), 1336–1343. 10.1212/WNL.0000000000002542. Epub 2016 Mar 4. 26944272 PMC4826337

[brb33259-bib-0028] Tufekci, A. , & Kara, E. (2021). Relation of serum hepcidin levels and restless legs syndrome in chronic hemodialysis patients. Sleep & Breathing = Schlaf & Atmung, 25(2), 897–905. 10.1007/s11325-020-02209-8. Epub 2020 Oct 7. 33029690

[brb33259-bib-0029] Urrutia, P. , Aguirre, P. , Esparza, A. , Tapia, V. , Mena, N. P. , Arredondo, M. , González‐Billault, C. , & Núñez, M. T. (2013). Inflammation alters the expression of DMT1, FPN1 and hepcidin, and it causes iron accumulation in central nervous system cells. Journal of Neurochemistry, 126(4), 541–549. 10.1111/jnc.12244. Epub 2013 Apr 3. 23506423

[brb33259-bib-0030] Weinstock, L. B. , Walters, A. S. , & Paueksakon, P. (2012). Restless legs syndrome–theoretical roles of inflammatory and immune mechanisms. Sleep Medicine Reviews, 16(4), 341–354. 10.1016/j.smrv.2011.09.003. Epub 2012 Jan 17. 22258033

